# Effect of Rural Trauma Team Development on the Outcomes of Motorcycle Accident–Related Injuries (Motor Registry Project): Protocol for a Multicenter Cluster Randomized Controlled Trial

**DOI:** 10.2196/55297

**Published:** 2024-05-07

**Authors:** Herman Lule, Micheal Mugerwa, Robinson Ssebuufu, Patrick Kyamanywa, Till Bärnighausen, Jussi P Posti, Michael Lowery Wilson

**Affiliations:** 1 Injury Epidemiology and Prevention (IEP) Research Group Turku Brain Injury Centre, Department of Clinical Neurosciences Turku University Hospital and University of Turku Turku Finland; 2 Department of Surgery Mengo Hospital Kampala Uganda; 3 Mother Kevin Postgraduate Medical School Uganda Martyr's University Nkozi Uganda; 4 Heidelberg Institute of Global Health (HIGH) University Hospital and University of Heidelberg Heidelberg Germany; 5 Neurocentre Department of Neurosurgery and Turku Brain Injury Centre Turku University Hospital and University of Turku Turku Finland

**Keywords:** randomized controlled trial, medical education, trauma team, trauma registry, rural health, global health, team development, Africa, rural, trauma, motorcycle injury, multicenter cluster randomized controlled clinical trial, injury, accident, low- and middle-income countries, patient outcomes, education program

## Abstract

**Background:**

Injury is a global health concern, and injury-related mortality disproportionately impacts low- and middle-income countries (LMICs). Compelling evidence from observational studies in high-income countries shows that trauma education programs, such as the Rural Trauma Team Development Course (RTTDC), increase clinician knowledge of injury care. There is a dearth of such evidence from controlled clinical trials to demonstrate the effect of the RTTDC on process and patient outcomes in LMICs.

**Objective:**

This multicenter cluster randomized controlled clinical trial aims to examine the impact of the RTTDC on process and patient outcomes associated with motorcycle accident–related injuries in an African low-resource setting.

**Methods:**

This is a 2-arm, parallel, multi-period, cluster randomized, controlled, clinical trial in Uganda, where rural trauma team development training is not routinely conducted. We will recruit regional referral hospitals and include patients with motorcycle accident–related injuries, interns, medical trainees, and road traffic law enforcement professionals. The intervention group (RTTDC) and control group (standard care) will include 3 hospitals each. The primary outcomes will be the interval from the accident to hospital admission and the interval from the referral decision to hospital discharge. The secondary outcomes will be all-cause mortality and morbidity associated with neurological and orthopedic injuries at 90 days after injury. All outcomes will be measured as final values. We will compare baseline characteristics and outcomes at both individual and cluster levels between the intervention and control groups. We will use mixed effects regression models to report any absolute or relative differences along with 95% CIs. We will perform subgroup analyses to evaluate and control confounding due to injury mechanisms and injury severity. We will establish a motorcycle trauma outcome (MOTOR) registry in consultation with community traffic police.

**Results:**

The trial was approved on August 27, 2019. The actual recruitment of the first patient participant began on September 01, 2019. The last follow-up was on August 27, 2023. Posttrial care, including linkage to clinical, social support, and referral services, is to be completed by November 27, 2023. Data analyses will be performed in Spring 2024, and the results are expected to be published in Autumn 2024.

**Conclusions:**

This trial will unveil how a locally contextualized rural trauma team development program impacts organizational efficiency in a continent challenged with limited infrastructure and human resources. Moreover, this trial will uncover how rural trauma team coordination impacts clinical outcomes, such as mortality and morbidity associated with neurological and orthopedic injuries, which are the key targets for strengthening trauma systems in LMICs where prehospital care is in the early stage. Our results could inform the design, implementation, and scalability of future rural trauma teams and trauma education programs in LMICs.

**Trial Registration:**

Pan African Clinical Trials Registry (PACTR202308851460352); https://pactr.samrc.ac.za/TrialDisplay.aspx?TrialID=25763

**International Registered Report Identifier (IRRID):**

DERR1-10.2196/55297

## Introduction

### Background

Transport-related injuries are a global public health threat and are anticipated to rank fifth in the global burden of disease by 2030 [1]. Evidence suggests that 90% of the transport-related crash mortality burden disproportionately occurs in low- and middle-income countries (LMICs), such as those in Africa [1], despite concerns of underreporting [2]. The main causes of injury-related mortality in Africa are road traffic accidents [3], which have serious economic consequences. According to Ryan-Coker et al [4], despite high poverty levels and minimal access to insurance and social protection schemes in Africa, which imply out of pocket expenditure, road traffic accidents cost US $119-$178 per injury and US $486-$12,845 per hospitalization, resulting in a gross domestic product (GDP) loss of 0.8%-9%.

Uganda is one of the LMICs in Africa with a high road traffic injury burden [5]. Our recent studies showed that most fatal injuries in Uganda result from motorized 2-wheeler and car collisions, contributing to 52.6%-64.7% of orthopedic and traumatic brain injuries [6-8]. Further, the majority (74%) of Uganda’s population lives in rural areas [9], which could partly explain the high injury-related mortality rates. Research has shown that the risk of trauma-related deaths in rural areas increases with remoteness owing to health care disparities, such as large commute distances, scarce resources, delays in referral, and lack of skilled trauma care providers, as well as population-specific and contextual challenges, such as poverty and impassable road networks [10].

Uganda lacks universal access to trauma care and injury surveillance systems owing to limited health care funding. Thus, Uganda has made sluggish progress toward attaining the sustainable development goal (SDG3) for universal health care coverage [11]. As such, in terms of the level of specialty care, the ratio of surgeons to patients is 0.7:25,000 as interns and medical trainees represent the largest proportion of Uganda’s health workforce [12,13].

Research has shown than more than half of the trauma patients in Uganda do not receive first aid prior to hospital arrival [6,7]. Moreover, most of these patients arrive at dedicated trauma centers much later than the recommended limit of 1 hour, and they are transported by either public vehicles, such as motorcycles [8], or police vans, without any focus on key factors, such as changes in blood pressure and oxygen circulation, which could lead to death, during transit [7,14]. These aspects have 3 important implications. First, strengthening the capacity for injury care among lay frontline workers. Second, stabilizing patients at rural regional trauma centers prior to transfer. Third, ensuring closed-loop communication between primary (level 3) and tertiary (level 1) trauma centers to enable safely coordinated referrals and adequate preparation at the receiving hospitals prior to patient transfer.

The key challenge that needs to be addressed in Ugandan rural settings is the lack of a formal prehospital care system as evidenced by emergency evacuation and transfer of trauma patients by untrained police staff [14]. Further, there is an immense need to redress the weak immediate care response after an accident as medical trainees are the first point of contact after an accident as opposed to specialists [12]. We will attempt to fix these challenges at the community level and at level 3 rural trauma centers by creating and providing training capacity for rural trauma networks between traffic police and medical interns or trainees at regional referral hospitals.

### Rationale

Evidence from 2 systematic reviews has shown that trauma education programs have the potential to improve skill acquisition and knowledge retention among health professionals; however, with regard to LMICs, most of these programs have not been locally contextualized and have been largely assessed based on their theoretical merit rather than their clinical impact on patients [15,16]. A recent observational study in Portugal showed that the European trauma course (ETC) improved self-efficacy and organization skills in individual routine practice, but the authors mentioned that future investigations should be conducted to examine the effect of the training on trauma outcomes [17]. This European study and systematic reviews in LMICs have recommended high-caliber epidemiological studies to evaluate the effects of such educational programs on process outcomes, such as organizational efficiency, and on patient outcomes, such as morbidity and mortality [15,16,18].

Except for an ongoing pilot trial comparing a primary trauma course (PTC) to advanced life support (ATLS) and routine care in India [19], trauma education programs in LMICs have mostly been evaluated based on nonrandomized studies, limiting their clinical uptake [15]. Moreover, most programs limit the course participants to qualified hospital-based medical providers [18], although interns, medical and allied health trainees, and road traffic law enforcement professionals are the most readily available frontline workers for managing injured patients in LMICs [14]. Inclusive planning while leveraging services that have no trauma designation, such as police services, is critical for expanding and strengthening complex rural trauma systems to improve injury reporting [2].

Locally contextualized trauma training has been identified as one of the most crucial steps for operationalizing nonfunctional rural trauma networks [10]. However, in a scoping review by Brown et al [18], only 12 out of 34 trauma courses in LMICs had been contextualized to suite low-income settings. Moreover, a cautiously executed integrative literature review on the challenges faced by trauma systems recommended that the next step for future research should be an examination of how trauma training impacts the outcomes of patients in remote environments [10]. This multicenter cluster randomized controlled trial will address this gap by examining the effect of the locally contextualized Rural Trauma Team Development Course (RTTDC) of the American College of Surgeons [20] on process and patient outcome measures in Ugandan trauma centers. The trial will focus on musculoskeletal and neurological injuries, which have been recently identified as the most important targets for strengthening health systems in rural Uganda [8], and it has been developed in accordance with the SPIRIT (Standard Protocol Items: Recommendations for Interventional Trials) guidelines for reporting outcomes in trial protocols [21].

### Objectives

#### Broad Objective

The main objective of this trial is to determine the effect of RTTDC training on the time efficiency of the clinical process and the patient-centered outcomes of motorcycle accident–related injuries.

#### Specific Objectives

The specific objectives are to (1) determine the effect of RTTDC training on the time interval from the accident to hospital admission and the time interval from the referral decision to hospital discharge (primary outcomes) and (2) determine the effect of RTTDC training on the outcomes of neurological and musculoskeletal injuries (secondary outcomes).

### Null Hypothesis

The null hypothesis of this trial is that RTTDC training has no effect on the time efficiency of the clinical process and the outcomes of orthopedic and neurological injuries.

## Methods

### Trial Design

This will be a pragmatic, 2-arm, parallel, multi-period, cluster randomized, controlled, clinical trial with 1 intervention (RTTDC) arm and 1 control (standard care) arm. The trial was set to start in 2019 but was delayed owing to the COVID-19 pandemic, and the subsequent prioritization of COVID-19–related studies by trial registers and target journals led to the delayed publication of this protocol.

This trial will embrace simultaneous cluster randomization, which is an ideal method for evaluating community-level interventions and overcoming ethical constraints that make randomization at the individual level impractical while avoiding probable contamination between the control and treatment groups [22]. Moreover, simultaneous randomization will enable timely operationalization and concurrent participant enrollment as all the outcomes will be measured as final values and there are no foreseen plans for study modifications in accordance with Esserman et al [23]. This arrangement is based on the fact that our previous studies have already provided insights on the likely rates of loss to follow-up and baseline mortality in our target trauma population and have informed the feasibility and relevance of collecting the intended primary and secondary outcomes [6,7].

### Study Setting

This study will be conducted in 6 specialized teaching and regional referral hospitals in Uganda, including Kiryandongo, Jinja, Hoima, Fort Portal, Mubende, and Kampala International University Hospital. These facilities have similar characteristics and serve as teaching, residency, and internship sites for undergraduate and graduate medical doctors and nurses. The hospitals offer 24/7 emergency surgical services for trauma patients through multidisciplinary teams of orthopedic, general, and visiting neurosurgeons, as well as imaging, rehabilitation, and physiotherapy. Each surgical department in these facilities is typically composed of about 1-4 faculty members, 2-4 specialty residents often referred to as senior house officers, 4-6 interns, and 10-30 undergraduate medical trainees.

These facilities are suited for this study as they serve both rural populations and populations in newly created populous cities that are still struggling with urban planning. Uganda is the 8th most populous country in Africa and the 30th most populous country in the world [9], and its trauma surveillance systems are in the infancy stage with weak preaccident and postaccident responses [6,12]. Despite efforts made in the past decade by the Ugandan government to address the unmet need for trauma care through infrastructure development, such as equipping operating theaters and intensive care units (ICUs) in public hospitals, limited standardized preservice and in-service trauma training, prehospital delays, and human resource constraints still contribute to preventable trauma mortalities [14]. To guarantee the quality of data for this project, a prospective motorcycle trauma outcome registry (MOTOR) will be piloted at the participating 6 regional referral hospitals in parallel with this trial.

### Eligibility Criteria

#### Inclusion Criteria

##### Study Sites

We will include level 3 trauma centers that are teaching hospitals; are staffed with medical trainees, interns, surgery residents, and consultants; and offer 24/7 emergency surgical care with access to blood banks, ultrasound scans, X-ray scans, and computed tomography (CT) scans as locally available or outsourced services.

##### Trainee Participants

We will include third-year or fifth-year medical or allied health students, interns, or specialty residents in surgery and traumatology clinical rotations who stay at the hospital of attachment for at least 2 months. Medical students may only rotate to a different hospital without crossing the study arm. In addition, we will include road traffic law enforcement professionals concerned with the evacuation of trauma patients from accident scenes.

##### Patient Participants

We will include patients who sustain motorcycle accident–related injuries and present to the study sites within 24 hours following the accident, as defined in a previous study [6]. These will include passengers on motorcycles, motorcycle riders, pedestrians or cyclists hit by motorcycles, and patients experiencing motorcycle-motorcycle collisions, motorcycle-static object collisions, or motorcycle-car collisions.

#### Exclusion Criteria

##### Study Sites

We will exclude trauma centers that do not offer placements and teaching facilities for students, interns, and residents.

##### Trainee Participants

We will exclude medical students who have not commenced their surgery clinical rotation and those not directly involved with the care of trauma patients at the time of the training, as trainee medical participants are expected to have already been introduced to surgery, emergency trauma resuscitation concepts, and trauma clinical scenarios through clerkships onto which we will be building the rural trauma team concept.

##### Patient Participants

We will exclude pregnant women, neonates, and infants aged 0-23 months owing to the known teratogenic effects of radiation in this population, as trauma evaluation in this study will involve obtaining radiographs for orthopedic injuries and CT scans for neurological injuries. Patients with documented stroke will also be excluded, as the study outcomes involve assessment for functional, physical, and neurological disabilities directly attributable to trauma. Moreover, mentally incapacitated patients who have no legally authorized representatives to sign an informed consent form and patients who die before hospital arrival or before imaging results are obtained will be excluded. Patients who are passengers in a car or drivers in a car at the time of the accident will be excluded, as the protective casing of the car body would make these patients less vulnerable to direct impacts compared with pedestrians, cyclists, or passengers on motorcycles. In addition, elderly patients older than 80 years will be excluded owing to their increased risk of fragility fractures that could misrepresent the severity of trauma. This cutoff age was used based on a systematic review by Tsuda et al [24], which documented a mean age of 80 years for long bone fragility fractures owing to increased muscle weakness, balance disorders, visual impairment, and dementia that predispose elderly people to falls. The number of ineligible participants and the reasons for their exclusion will be recorded in both arms in the consolidated standards of reporting trials flow diagram.

### Ethical Considerations

#### Research Approval

Prior to recruitment, this study has been approved and registered by the research and ethics committees of the Uganda National Council for Science and Technology (reference number: SS 5082) and Mbarara University of Science and Technology (reference number: MUREC 1/7; 05/5-19).

#### Consent to Participate

Written informed consent will be obtained from all study participants or their legally authorized representatives prior to participation. The official informed consent form documents for Mbarara University of Science and Technology will be adopted for this purpose. Both trainee and patient participant informed consent documents are provided in Multimedia Appendix 1 and Multimedia Appendix 2, respectively. The ethical committees ruled that trainees involved in the data collection process at control centers do not need to provide any consent as this study will neither directly affect their practices nor collect their personal data.

#### Collection of Informed Consent

All participants or their legally authorized representatives (for minors and unconscious patients) will endorse a predesignated consent form document with their signatures in the presence of the principal investigator or research assistants (surgery specialty residents).

#### Additional Consent Provisions for the Collection and Use of Participant Data and Biological Specimens

There will be no biological specimens retained for this study. The informed consent form documents will have provisions for consenting to follow-up and the use of data for approved ancillary studies, and for permission to archive the anonymized data in a public data repository.

#### Consent for Publication

This manuscript does not contain any identifying individual participant information or images; thus, consent for publication is not applicable.

#### Privacy and Confidentiality Protection

To protect participants’ confidentiality before, during, and after the trial, all hard copy data collection items will be kept under lock and key and will bear unique nonidentifying codes. Soft copies will be kept in password-protected computers, with a second-layer protection login password required for REDCap, which will be only accessible to the study team. The final data sets will be anonymized prior to archiving and publication. Hard copies of the data will be destroyed 6 months after completion of the trial.

#### Compensation Type and Amount for Participants

Participation in this trial will be voluntary. However, transport reimbursement of 20,000 Ugandan shillings (US $5.0) will be provided to participants turning up for research follow-up appointments outside of their routine hospital visits. Moreover, time compensation of 10,000 Ugandan shillings (US $2.5) will be provided.

### Interventions

#### Explanation for the Choice of Comparators

The intervention for this trial is based on the fourth edition of the RTTDC of the American College of Surgeons [20], which will be delivered to medical trainees, interns, and traffic law enforcement professionals at the 3 intervention study centers (intervention group). Specialty surgical residents will be trained as faculty who will later serve as trainers since they directly supervise interns and undergraduate medical students in Ugandan settings. The research and ethics committees approved training residents as opposed to specialists to avoid constraining the scarce specialized human resource as this is a trainee-capacity building trauma education program. Moreover, consultants rarely attend to trauma patients for immediate resuscitation as the first point of contact in Ugandan settings. The patient participants at intervention sites will be those with motorcycle accident–related injuries at any of the study sites. The comparator in this trial is a control group of hospitals that will offer standard care to eligible patients, without their care providers receiving the RTTDC training intervention.

#### Intervention Description

The 2-day RTTDC interventional training will be conducted in designated spacious multimedia surgical simulation conference rooms at the study sites randomized to the intervention arm and will be delivered in the described standard format [20]. This training model is the most appropriate for Uganda, with its health worker to patient ratio of 1:25,000 and its large population of 48 million [12]. The model dwells on team concepts to improve coordination and efficiency of the existing “skeletal” health structure in the rural environment. We hope to train a total of 66 road traffic police officers, 12 specialty residents, 30 intern doctors, 140 fifth-year medical students, and 264 third-year medical students. These figures have been determined based on the average annual number of traffic police officers and trainees at the respective regional police headquarters and hospitals [25].

Our target is to train at least 80% of each cohort of eligible trainees identified every 1-5 months within the surgery department during the 4-year study period until the required trainee and patient participant sample sizes are attained. If trainees drop out prior to completion of data collection, compensations will be made to maintain the criterion during the next cohort of surgical rotation trainee intake. The details of how the training will be conducted have been provided elsewhere [25]. The research core team has agreed that trainee participants who score more than 60% in posttraining trauma-based multiple-choice questions (MCQs) at the intervention sites will be retained to form a rural trauma team network, which will constitute multiple local 6-member trauma committees of first responders with defined roles (ie, each team having 5 medical trainees and 1 contact road traffic officer).

**Figure 1:**
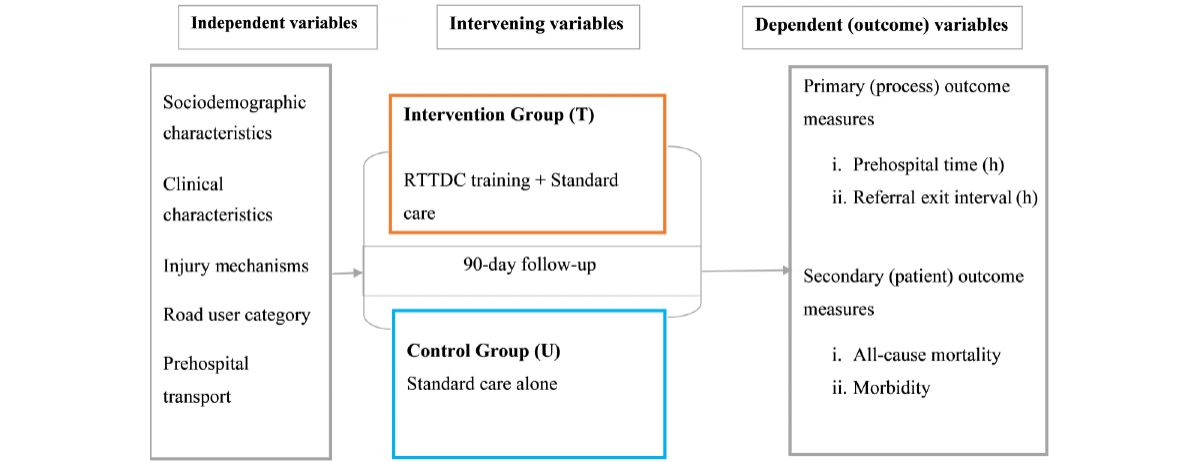
Conceptual framework summarizing the intervention and the interaction of variables. RTTDC: Rural Trauma Team Development Course.

To enable an active “alarm” activation criterion for the trauma committees, closed-loop communication, and a smooth “handover” process from police officers to medical trainees prior to patient transfer, the trained traffic officers on highways will serve as focal contact persons for the rural trauma teams from the parish (subcounty) level. In the event of a road traffic accident, teammates will freely contact each other both within and across teams to coordinate evacuation, consultation, referral, and arrival at recipient centers, with the support of trained interns and surgery residents as team leaders. Weekly audit meetings will be conducted remotely via Zoom (Zoom Video Communications) to discuss team challenges, otherwise a trained surgery resident will be available 24/7 to advise on referrals, transfers, and treatments in liaison with consultants who may be physically or remotely available. The control hospitals in this trial will be allowed to provide routine standard care without the RTTDC intervention as summarized in Figure 1.

#### Criteria for Discontinuing or Modifying the Allocated Intervention

The fourth edition of the RTTDC will be delivered in its standardized form [20] without any modifications.

#### Strategies to Improve Adherence to the Intervention

The RTTDC is the intervention; thus, participants who do not attend all the modules and complete the posttraining MCQs will be excluded. Adherence to teachings in the RTTDC among care providers during clinical practice, such as the time taken from referral decision to execution and improper closed-loop communication leading to loss to follow-up, will be assessed as process measure outcomes.

### Relevant Concomitant Care Permitted or Prohibited During the Trial

The baseline quality of care given to trauma patients at each of the participating 6 hospitals is based on the regulations of the Uganda Medical and Dental Practitioners Council (UMDPC), which regulates medical practice, and the National Council for Higher Education (NCHE), which regulates both undergraduate and graduate surgical curricula [26]. The councils require that graduating doctors undergo clinical rotations in surgery and traumatology, perform clerking activities, and present and document trauma scenarios in patient case files.

In addition, medical graduates should demonstrate the ability to initiate emergency resuscitation in accordance with advanced trauma life support protocols [27], such as execution of primary and secondary surveys; carefully discern, request, and comprehend the necessary imaging and laboratory investigations in trauma; acknowledge self-knowledge and resource limitations; and consult or refer patients whose surgical care demands exceed their own skills or the local trauma capacity. Further, upon graduation, Ugandan doctors are required to obtain a minimum of 48 hours of continuous professional development points annually to maintain their licensure.

In terms of the standards of care, both intervention and control facilities have medical trainees, interns, specialty residents, and specialists who provide care for injured patients. The care typically involves receiving trauma patients who may be brought by staffed ambulances, police officers, or public means to accident and emergency units, and these patients may be received by trainees, interns, or surgical residents. These cadres open case files; initiate immediate care through horizontal consultation; request X-ray scans, CT scans, and laboratory workup; and plan definitive care or referral in liaison with a senior surgical resident and in consultation with a multidisciplinary team of specialists on call. As such, procedures, such as surgical suturing and toileting, limb fracture casting and immobilization, and chest tube insertion, are often performed by interns. More demanding procedures, such as splenectomy, external fixation of fractures, and burr holes, are usually performed by specialty residents after notification of the consultant on duty, whereas major cases of polytrauma warrant the decision of the consultant specialist on call before the patient is taken to the operating room.

Imaging and blood transfusion services are often freely available at emergence units as government services but may be outsourced from private hospitals at a cost owing to long waiting times or service maintenance for CT scanners and X-ray machines. Finally, in terms of patient disposition, patients with minor injuries requiring surgery under local anesthesia are discharged on the same day. Moreover, patients with major trauma requiring conservative treatment, such as closed fractures, are treated in surgical wards, whereas those who are deeply unconscious or require critical care after major operations are admitted to the ICU until they are fit for care in general surgical wards prior to discharge. Alternatively due to overcrowding, often with higher bed occupancy rates exceeding the predetermined bed capacity, patients with major trauma may be referred to other centers, and the common reasons for referral include further neurosurgical evaluation with magnetic resonance imaging or further evaluation by a neurosurgeon, need for multiple specialty care and rehabilitation, and need for ICU admission elsewhere when local ICU beds are limited.

### Provisions for Posttrial Care

Any participants requiring further care beyond the 90-day follow-up will be linked to their attending clinicians during an additional 3-month period after completion of the trial. There will be no compensation other than transport reimbursement for participants visiting solely for study follow-up outside routine clinical visits as this study was considered minimal risk by the approving research ethics committees. The principal investigator will refund the cost of imaging when the request is approved by attending surgeons and radiologists in cases where the services are unavailable free of charge.

### Outcomes

This trial will examine both process measures and patient outcomes. The primary (process) outcomes of this trial will involve comparisons of (1) the time interval from the accident to hospital admission and (2) the time interval from the referral decision to hospital discharge between patients with motorcycle accident–related injuries presenting to the intervention centers and those presenting to the control centers.

The secondary (patient) outcomes of this trial will involve comparisons of (1) all-cause mortality at 90 days from the time of injury and (2) morbidity of motorcycle accident–related orthopedic and neurological injuries between patients presenting to the intervention centers and those presenting to the control centers. The morbidity of orthopedic injuries will be measured based on the Trauma Expectation Factor Score (TEFS) at admission and the Trauma Outcome Measure Score (TOMS) at 90 days as reported to outcome assessors [28]. On the other hand, for neurological injuries, the Glasgow Coma Scale (GCS) at admission and Glasgow Outcome Scale (GOS) at 90 days will be used as documented by the attending clinicians [29].

The tertiary outcomes will involve the effects of the training on provider knowledge based on pretraining and posttraining trauma-related MCQ scores and the barriers to injury care faced by providers during execution of definitive treatment. These will be determined in 2 ancillary studies that have been approved as part of this trial, and the protocols have been detailed elsewhere [25]. A summary of all outcomes and their case definitions, specific measurement variables, analysis metrics, methods of aggregation, and time points has been provided in Multimedia Appendix 3.

The patient outcomes were selected based on their validity and relevance in previous studies [7,30]. Further, level III evidence suggests that prolonged prehospital times may be associated with increased mortality among trauma patients [31]. However, it is unclear how these results relate to time-sensitive orthopedic and neurological injuries in LMICs that lack formal coordinated prehospital systems [32]. Thus, our process outcome measure variables were selected within the context of evidence-based practice. This practice stipulates that in accordance with the golden hour rule, a major trauma patient should be in the right place within 60 minutes following injury, otherwise there is a risk of mortality or morbidity [33].

These variables align well with the proposed global surgery framework to improve national surgical, obstetric, and anesthesia care provider plans [34]. In addition, the variables expand on outcomes for trauma-informed interventions [35]. All outcomes will be measured as final values but will be discussed in relation to the baseline established in our prior feasibility studies [7,30]. The comparisons of outcomes will be made at both individual and cluster levels, using mixed effects regression models.

### Participant Timeline

Initially, cluster randomization of the trauma centers will be performed 3 weeks prior to commencement of the training to determine which centers will receive the training. Potential participants for the training will be screened for eligibility by the hospital and study administrators 2 weeks prior to the training, and eligible trainee participants will be consented and assigned to “rural trauma teams” of 6 members on the first day of the training, during which they will complete a pretraining questionnaire involving trauma-based MCQs. Subsequently, trainee participants will be followed up at 90 days (3 months) to complete posttraining MCQs. The first eligible patient participants at both control and intervention sites will be enrolled concurrently through a motorcycle trauma outcome registry (MOTOR) that will run parallel to the trial.

Informed consent, baseline sociodemographic and clinical characteristics, and prehospital intervals will be obtained at admission, and the GCS score will be determined. The TEFS will be obtained during the first week of admission or prior to discharge on the assumption that patients would have received their definitive surgical intervention or would have been referred for further care during this period. Further, the GOS score, all-cause mortality rate, and TOMS will be obtained at the 90-day follow-up. The GCS will be used to assess the level of traumatic brain injury in the acute phase, whereas the GOS will be used to determine neurological outcomes within the context of the level of independence at a later phase of traumatic brain injury [29]. On the other hand, the TOMS will be used to assess patient-reported trauma outcomes with reference to their expectations (TEFS) at the time of the trauma intervention in relation to the levels of pain, physical function, disability, injury treatment satisfaction, and overall satisfaction [28,30]. Throughout the study period, any individual patient barriers encountered during the pathway to execution of definitive injury care will be documented as summarized in the timeline presented in Multimedia Appendix 4.

### Sample Size

To estimate the sample size, we used the open-source R Shiny application for cluster randomized controlled trials with a parallel design and discrete time decay correlation structures for multiple periods [36] available at [37]. Further, we assumed *t*-distribution due to plans for small sample corrections at the analysis stage in accordance with the report by Rutterford et al [38]. The study was approved for 4 years, but the core research team foresaw it feasible to actively collect data for a period of 3 years considering university semester breaks, public holidays, and unexpected events, such as COVID-19–related interruptions, which led to suspension of the trial for 12 months from March 2020 to March 2021. The 3 years (36 months) would yield 12 study periods given the planned training of cohorts of medical students on a 3-monthly basis in accordance with the average duration of internship deployment by the Ugandan Ministry of Health (1-5 months) and the average duration of surgery clinical rotation for university medical students (2 months).

**Figure 2:**
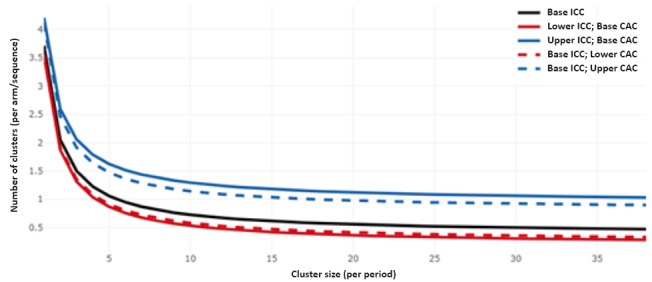
Reduction in the number of clusters required with an increase in the cluster-period size at a fixed power of 0.8. CAC: cluster autocorrelation coefficient; ICC: intraclass correlation coefficient.

Fixing the study power at 80% for a parallel cluster randomized trial with different cross-sections, considering a discrete time decay period of every 3 months for a total of 12 periods, and assuming correlations to decay with each period, we explored the minimum number of patient participants (cluster size) required per period using the R Shiny application [36]. Assuming a significance level of .05, a within-period intraclass correlation coefficient (ICC) of 0.02 (lower extreme: 0.01, upper extreme: 0.05), and a cluster autocorrelation coefficient (CAC) (ratio of between-period ICC to within-period ICC) of 0.8; allowing for varying cluster sizes with a coefficient of variation of 0.5; and considering a mean difference of 1.02 hours in prehospital transfer time and a pooled standard deviation of 1.64 hours as continuous outcomes reported in a previous observational study from the United States [39], the upper ICC and base CAC plateaued between 5 and 10 participants (Figure 2).

**Figure 3:**
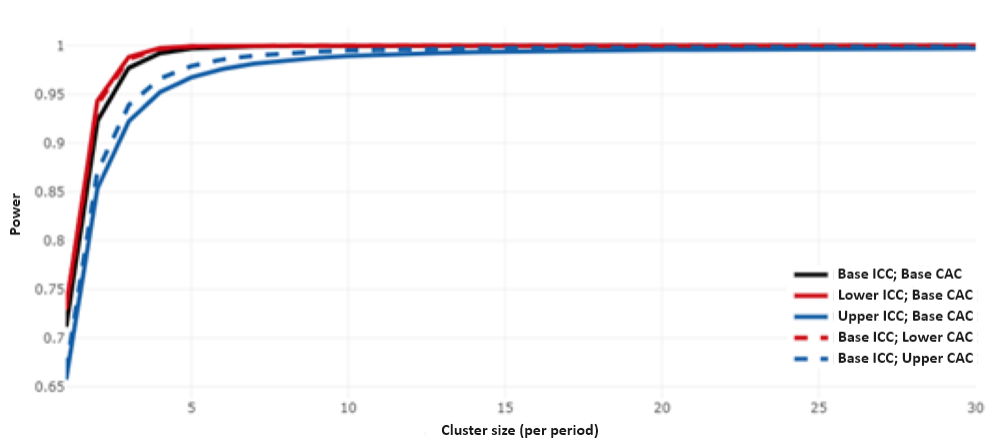
Cluster-period size of 10 meeting the required power of 0.8 for 12 periods. CAC: cluster autocorrelation coefficient; ICC: intraclass correlation coefficient.

**Figure 4:**
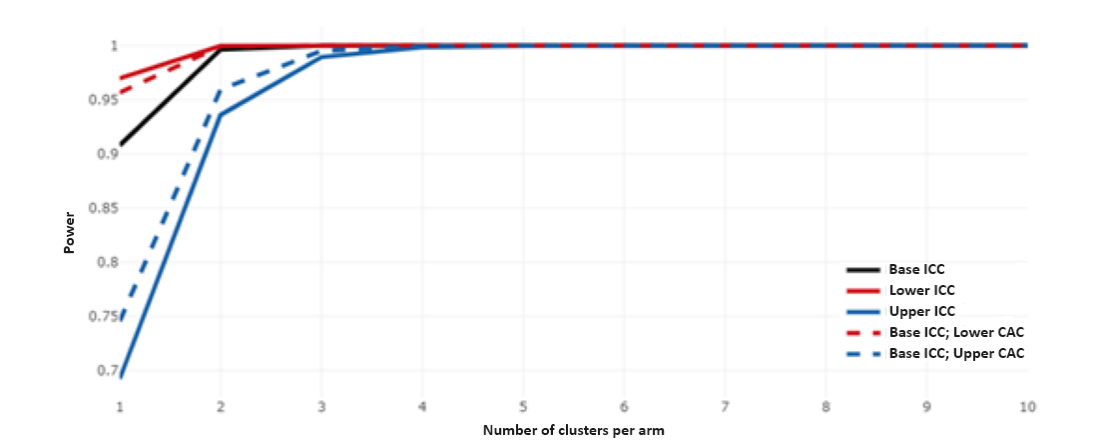
Three clusters per arm meeting the minimum required power of 0.8 for 12 periods. CAC: cluster autocorrelation coefficient; ICC: intraclass correlation coefficient.

Considering a maximum of 10 participants per cluster period, an 80% power could be attained (Figure 3) and could be met with a total of 3 clusters per arm (Figure 4). This indicates a total of 6 study centers (clusters) drawn from a pool of 17, which reasonably represents 35.3% of Uganda’s regional referral hospitals.

The sample required per arm is calculated as follows: 10 participants per cluster period multiplied by 12 cluster periods multiplied by 3 clusters, yielding a total of 360 participants. The total sample required for 2 arms would be 720. However, we inflated the sample and variance to cater to the clustering design effect based on the assumption that this increases the statistical power in accordance with the repost by Hemming et al [36]. Thus, the derived sample size (s) is calculated using the following formula:

s = n (1 + [m − 1] ρ) **(1)**

where s is the required sample size, m is the cluster size per period (ie, 10), and ρ is the within-period ICC (0.02).

This formula yields a total of 850 participants.

We will add a rate of loss to follow-up of 18% assuming the worst scenario based on our previous dropout rate of 9% in a feasibility study [7]. Therefore, the final sample will include approximately 1003 participants. Assuming an equal allocation ratio of 1:1, there will be 502 participants per arm and about 168 individuals per cluster. This sample is deemed feasible considering the high trauma burden in Uganda. According to a study at one of the Ugandan rural regional referral hospitals, a total of 900 motorcycle accident–related injuries were recorded during a 3-year period, of which 30.9% (278/900) and 15.8% (142/900) of patients sustained musculoskeletal and neurological injuries, respectively [8]. We will not compute sample sizes for each individual secondary outcome as previous studies have not reported any difference in mortality [40].

### Recruitment

The training will be advertised by hospital administrators, class representatives, and regional traffic law enforcement leaders, and course participants will be recruited by the principal investigator and the respective hospital administrators. The patient participants will be recruited by dedicated project officers at the accident and emergency departments.

### Assignment of Interventions: Allocation

#### Sequence Generation

We will perform cluster randomization for a permuted block size of 6 hospitals (clusters) using an open-source simulation software [41], with seed numbers that will be kept confidential to the offsite study administrator. A list of 6 random codes will be generated to assign hospitals to the intervention or control group. As we plan to perform analyses at both the individual level and treatment arm level, we will not add any stratification factors in the simulation model.

#### Concealment Mechanism

The allocation sequence and assignment codes will be generated by and kept secret by an offsite study administrator. Both outcome assessors and patient participants will be blinded to the treatment allocation.

#### Implementation

Trainee participants will be enrolled by the respective university hospital administrators, whereas patient participants will be enrolled by project medical officers at the respective study sites, who will serve as outcome assessors.

### Assignment of the Intervention: Blinding

#### Who Will Be Blinded

Trial patient participants and outcome assessors will be blinded using blocked allocation sequence codes.

#### Procedure for Unblinding if Needed

Unblinding will only be permissible in the likely occurrence of adverse events among the study participants, which can be directly attributed to the study intervention. Such events will be discussed with the attending physicians. Otherwise, the allocation codes will only be revealed to the biostatistician at the time of interim analysis when half of the sample size is recruited. At this point, unblinding the biostatistician is considered more beneficial to inform termination or continuation of the trial, which outweighs the perceived fear of unknown bias [42].

### Data Collection and Management

#### Plans for Assessment and Collection of Outcomes

Data collection will start simultaneously at all the study sites. Medical officers (blinded outcome assessors) with a minimum qualification of Bachelor of Medicine and Bachelor of Surgery (MBChB) and at least 2 years of clinical experience will prospectively collect data through clinical observations, interviews, home visits, and extraction from hospital and police records. All outcome assessors must be willing to undertake a 1-day budgeted training in the use of data collection tools for this trial and the World Health Organization (WHO) ICD-10 online coding module. The data will be collected daily as medical trainees and interns will attend clinical rotations every day. Upon obtaining informed consent, the venue for data collection, including follow-up information, will be at accident and emergence departments during admission, surgical wards, and outpatient clinics, as well as through home visits and phone calls to patients, care takers, or recipient hospital physicians in the case of referrals.

The study variables will include sociodemographic and clinical variables, such as sex, age, injury mechanisms, including nature of collision and road user category, time of injury, prehospital transit time, prehospital care, mode of arrival, vital signs, nature of physical injuries, radiological findings, and injury severity based on the GCS score and Kampala Trauma Score (KTS) [43]. In addition, data on the nature of the treatment administered; need for referral; referral-exit interval; and outcomes, including the TOMS, GOS score, mortality, and time from injury to death, will be obtained. The data collection tools that will be used in this trial, such as the GCS, GOS, TEFS, TOMS, and KTS, have been shown to demonstrate excellent criterion internal validity, consistence, and reproducibility in previous studies [28,29,43,44].

Further, the tools are recognized for their high interrater reliability, sensitivity to change, and ability to be used as continuous or categorical variables in validation studies [28,29,43,44]. The baseline TEFS, GCS score, and KTS will be added as covariates whose interaction and confounding will be assessed and controlled since the inclusion of baseline values of outcomes as covariates is arguably one of the strongest factors to reduce ICC estimates [45]. The difference in distribution of baseline characteristics will be compared using means (SDs), medians (IQRs), and ranges for quantitative data; otherwise, frequencies and percentages will be used for categorical variables. All data collection forms for this trial and detailed definitions of assessment tools for patient and trainee participants are available in Multimedia Appendix 5 and Multimedia Appendix 6, respectively.

#### Plans to Promote Participant Retention and Complete Follow-Up

Coffee breaks will be facilitated during training to optimize participation. Phone and email contacts will be obtained from all medical trainees and law enforcement professionals. In addition, 2 phone contacts will be obtained for each patient participant at the time of enrollment at accident and emergency departments (ie, the patient and their next of kin or legally authorized representative who will in turn be provided with the phone contact of the study nurse coordinators for the purpose of follow-up). Automated reminders will be sent to data collection assistants through mobile phones and the research electronic data capture (REDCap) platform.

Where applicable, home visits will be conducted for participants who are unable to turn up for appointments because of their disability. Transport costs will be reimbursed for traffic law enforcement professionals and patient participants turning up for appointments outside of the routine hospital visits. Participants will be considered as lost to follow-up if they are untraceable by phone, clinic appointment, or home visit. Data on the baseline sociodemographic and clinical characteristics of those lost to follow-up or those who discontinue because of a breach of the intervention protocol will be retained for comparisons between groups, but the data of those who withdraw their consent will be deleted.

### Patient and Public Involvement

Using semistructured questionnaires and semistructured interviews, a 1-day consultative meeting will be conducted at each study site with chief residents, heads of interns, patient caregivers, student representatives, and regional traffic police commanders in the month preceding study commencement to discuss their perceived barriers to injury care in order to uncover themes for potential sources of delays ranging from accident scene discovery, evacuation, and prehospital transportation to emergency care, which shaped the final data collection tools.

During this engagement, trainee and caregiver representatives will provide insights on the feasibility of the outcome assessment tools and the time commitment required to respond to the questionnaire, which will inform the data collection time points of day 1, the first week, and day 90. This strategy will strike a balance regarding the feasibility of maximizing response rates and obtaining data while not overloading the already scarce human resources of traffic law enforcement, and trainees and clinicians at accident and emergency departments. Further engagements will be made during RTTDC training sessions and audit meetings, during which bidirectional feedback will be provided on team performance, challenges with individual team dynamics, and proposed areas of improvement to help shape future training. The results of patient and public engagement will be reported in an ancillary study on the barriers to injury care.

### Data Management

Due to expected network breakdowns in rural centers, data will be collected in hard copies, coded, and entered into the REDCap secure virtual network hosted by the University of Turku. Data entry will be performed by outcome assessors who will be issued with a confidential login password for REDCap. Later, the data will be exported to Stata version 15.0 (StataCorp) for analysis. The REDCap software was preferred owing to its presumed secure web-based intuitive interface for validated data capture, offering an additional advantage to prohibit dual entry, restrict values, calibrate data ranges, and retrieve audit trails for tracking data manipulation [46]. Further, the software permits seamless data export and download procedures that are compatible with Stata, while allowing for data integration and interoperability with external sources [47]. Any data errors will be resolved during weekly audit meetings in reference to the online codebook with a visible description of variables accessible to the principal investigator and all outcome assessors.

### Plans for Collection, Laboratory Evaluation, and Storage of Biological Specimens for Genetic or Molecular Analysis

There are no plans to collect any biological laboratory specimens for storage or for genetic or molecular analysis in this trial.

### Statistical Methods

#### Statistical Methods for Primary and Secondary Outcomes

##### Primary (Process) Outcomes

The primary (process) outcomes of (1) the time interval from the accident to hospital admission and (2) the time interval from the referral decision to hospital discharge will be compared between the intervention and control groups using a 2-sample *t* test if the data are normally distributed or a 2-sample Wilcoxon rank sum test if the data are not normally distributed. The normality of distribution will be assessed using the Shapiro-Wilks test, whereas the equality of variance will be determined using the Levene test. A difference in means of 60 minutes (1 hour) will be considered clinically meaningful in accordance with the golden hour principle [33].

##### Secondary (Patient) Outcomes

For assessing the morbidity of musculoskeletal injuries, the validated 10-item TEFS will be used at baseline and the 10-item TOMS will be used at 90 days [30]. The mean difference in the TEFS and TOMS will be compared between the intervention and control groups using the 2-sample *t* test. Further, the TOMS will be dichotomized into favorable (TOMS ≥ TEFS) and unfavorable (TOMS < TEFS) outcomes in accordance with previous studies [30]. The difference in these proportions between the intervention and control groups will be compared using the adjusted chi-square test if the expected count is >5 or the Fisher exact test at 95% CI otherwise.

Further, subgroup analyses will be performed to determine and control for factors that could be associated with an unfavorable TOMS, using individual-level between-within mixed effects regression models, which inherently overcome the effect of smaller clusters and permit adjustment for covariates in a single stage, assuming equal allocation. The fixed effect variables will be the treatment arms (intervention vs control) as the unit of analysis, with the odds ratios (ORs) and their corresponding 95% CIs as direct estimates of the effect size. The covariates will include age, sex, education level, occupation, employment status, marital status, commute distance, road user category, injury severity score based on the KTS, presence or absence of fracture, nature of fracture if present, number of serious injuries, and treatment (surgical vs conservative).

For assessing the morbidity of neurological injuries, the validated GOS will be used at 90 days after injury. The mean difference in the GOS score will be compared between the intervention and control groups using the 2-sample *t* test. Further, the GOS score will be dichotomized into favorable (GOS score of 4 or 5) and unfavorable (GOS score of 3, 2, or 1) outcomes in accordance with previous studies [7]. The difference in proportions between the intervention and control groups will be compared using the adjusted chi-square test if the expected count is >5 or the Fisher exact test at 95% CI otherwise.

For all-cause mortality, the difference in proportions of all deaths at 90 days after injury will be compared between the intervention and control groups using the chi-square test if the expected count is >5 or the Fisher exact test at 95% CI otherwise. Lastly, we will conduct subgroup analyses to determine and control for factors associated with all-cause mortality using individual- and cluster-level between-within mixed effects regression models. The covariates will include age, sex, injury mechanism (including helmet use), mode of arrival, prehospital interval, referral decision to hospital discharge interval (dichotomized as ≤1 hour or >1 hour), prehospital first aid status, comorbidities, injury severity score based on the GCS at baseline, multiplicity of injuries, head and brain CT-based diagnosis, and neurosurgical intervention.

These variables have been found to influence outcomes following neurological trauma in previous studies [7]. All variables will be analyzed for confounding and effect modification using Mantel-Haenszel statistics to probe necessary interaction terms. Variables with *P* values of ≤0.2 in the bivariate analysis will be included in the multivariate analysis. The median (IQR) of the time to the event, that is, from the accident to death (in days), will be stratified by treatment arm, and the difference will be compared using the Wilcoxon 2-sample test. All analyses will be performed using Stata 15.0, and where appropriate, graphics, such as box plots, will be used to visualize the data.

#### Interim Analyses

The Uganda National Council for Science and Technology accessed a preliminary report of the interim analysis, which was performed on August 28, 2022, when half of the sample size (n=500) was attained, and at its discretion, it recommended continuation of the study. The requisite characteristics for early termination set by the ethics committee included the occurrence of an index patient-reported adverse outcome definitely attributable to the study in accordance with the framework described by Okoniewska et al [48].

#### Methods for Additional Analyses

Subgroup analyses will be performed for (1) varying road user categories (pedestrian, passenger, and motorcyclist); (2) injury mechanisms (motorcycle-motorcycle accident, motorcycle-pedestrian accident, motorcycle-static object accident, and motorcycle-car accident); (3) varying injury severities (mild, moderate, or severe based on the KTS and GCS); and (4) multiplicity of serious injuries (one or multiple). To determine the training-effect heterogeneity across time periods, we will use an extension of Hussey and Hughes fixed effects model [49] for determining whether the intervention effect differs for each training period as summarized in the schematic representation of the study design (Multimedia Appendix 7).

For the ancillary study on provider outcomes, the difference in pretraining and posttraining mean scores will be computed with 95% CIs if the data are normally distributed; otherwise, the difference in median and IQR will be reported. For the qualitative ancillary study on the perceived barriers to injury care, directed content analysis of themes of transcribed data will be collated manually and presented as percentages.

#### Methods in Analysis to Handle Protocol Nonadherence and Any Statistical Methods to Handle Missing Data

We will impute values for participants with missing end points, such as those lost to follow-up, those who withdraw consent, and those who crossover. The baseline sociodemographic and clinical characteristics of such participants will be compared between the intervention and control groups. For individual participants, crossover from the intervention group to the control group or vice versa for any reason will result in ultimate discontinuation from the trial.

#### Plans to Provide Access to the Full Protocol, Participant-Level Data, and Statistical Code

The full protocol will be published open access, and anonymized participant-level data sets and statistical codes will be shared publicly through a permanent weblink that will be provided by the publishing journal. Since the primary country of recruitment, which approved and registered the study prior to recruitment, lacks a publicly available electronic research register, this protocol has been retrospectively registered with the WHO-approved Pan African Clinical Trials Registry (PACTR202308851460352).

### Oversight and Monitoring

#### Composition of the Coordinating Center and Trial Steering Committee

The principal investigator and a central study administrator will form a steering committee that will run the day-to-day activities of the trial. The trial will have 2 onsite overseers and 2 off-site supervisors. In addition, the principal investigator will provide organizational support to rural trauma teams at the intervention study centers through weekly audit meetings. Each of the rural trauma teams is composed of a road traffic law enforcement professional, a third-year medical student, a fifth-year medical student, an intern doctor or nurse, and a specialty surgery resident.

#### Composition of the Data Monitoring Committee, and Its Role and Reporting Structure

The research and ethics committee of Mbarara University of Science and Technology is the designated independent data monitoring committee that will oversee this trial (reference number: MUREC 1/7; 05/5-19). The committee reports directly to the Uganda National Council for Science and Technology. The council, at its discretion, can recommend continuation, amendments, or termination of the trial at any time.

### Adverse Event Reporting and Harms

Immediate medical concerns from participants will be reported to their attending clinicians. Any reported adverse events and other unintended effects of trial interventions or trial conduct will be reported to the trial monitoring committee. Patient-reported adverse outcomes resulting from the intervention (study) rather than biological injury progression will be captured during the follow-up interviews and evaluated on a 5-point Likert scale in accordance with the framework described by Okoniewska et al [48] as follows: (1) no evidence that the event is due to the intervention, (2) little evidence that the event is due to the intervention, (3) the event is possibly due to the intervention but most likely due to injury, (4) the event is more likely due to the intervention than injury, and (5) the event is definitely due to the intervention. Case files with 4 to 5 points will be forwarded for external auditing in surgery departmental meetings to ascertain if the adverse event was preventable, ameliorable, or neither.

### Frequency and Plans for Auditing Trial Conduct

The trial monitoring committees will independently conduct annual and impromptu audits and may choose to extend or terminate the trial at any time. Such situations that could warranty termination include adverse events directly attributable to the intervention. The investigators will submit annual progress reports to the committees each year as part of continuing review.

### Plans for Communicating Important Protocol Amendments to Relevant Parties

Any protocol amendments will be submitted to the data monitoring committee. Any approved amendments will be communicated to the Uganda National Council for Science and Technology and trial participants, and will be updated in the registries within 5 working days following approval.

### Ancillary Studies

This trial has been approved with 2 ancillary studies. The first is a study to assess the effect of the RTTDC on provider knowledge, and the second is a qualitative study to assess the barriers to injury care as perceived by traffic police, medical trainee frontline workers, and individuals encountered in the real-time management of patient participants. The data collection team for the qualitative study will be surgery residents at the 6 regional referral hospitals who will be blinded to the cluster or treatment allocation. The results of the ancillary studies will be collected and analyzed separately and will be concealed from the trial team until the analysis of the main clinical trial findings is nearly complete. We hope that the findings of the ancillary studies will complement and inform the interpretation of the trial findings.

### Dissemination Plans

Participants of the RTTDC training will receive feedback after the posttest questionnaire evaluation. Patient participants will be advised on the next plans of management during follow-up calls or through outpatient consultations. Before presentation of the study findings at scientific conferences and publication in peer-reviewed journals, copies of the findings in final bound reports will be submitted to the main libraries of the participating hospitals, the departments of surgery, and the internal review boards of Mbarara University of Science and Technology, Kampala International University, and Uganda National Council for Science and Technology.

The implications of the findings will be shared with authorities, including intern and medical student associations, district health officers, hospital directors, regional traffic police commanders, and chairpersons of the motorcycle riders’ associations in the respective regions, as well as with the rural trauma networks of first responders. Priority will be made to present the preliminary findings at an international conference hosted in Uganda and at the annual world safety conference. Anonymized participant data sets from this trial will be archived on a publicly accessible permanent weblink that will be provided by the publishing journal within 12 months from the actual date of completion of the trial.

## Results

The trial was approved on August 27, 2019. The actual recruitment of the first patient participant began on September 01, 2019. The last follow-up was on August 27, 2023. Posttrial care, including linkage to clinical, social support, and referral services, is to be completed by November 27, 2023. Data analyses will be performed in Spring 2024, and the results are expected to be published in Autumn 2024.

## Discussion

### Study Implications and Future Directions

This trial is meant to compare (1) the time interval from the accident to hospital admission, (2) the time interval from the referral decision to hospital discharge, (3) the all-cause mortality, and (4) the morbidity of patients with motorcycle accident–related orthopedic and neurological injuries between RTTDC (intervention) and standard care (control) study centers in Uganda, Africa.

This study is anticipated to reveal the overall impact of the rural trauma team development and training on the clinical process time efficiency and patient-centered outcomes of musculoskeletal and neurological injuries in controlled clinical settings, using validated injury severity scores [43] and trauma outcome and process measures [29,30]. The findings will add value to the emerging evidence from previous nonrandomized studies in high-income countries, which have evaluated the RTTDC. The available evidence suggests that the RTTDC potentially reduces the prehospital interval [50] and referral decision time [50], and enhances trauma team role identity, but has no perceived impact on mortality reduction [51]. It remains unclear how these findings relate to LMICs whose trauma care systems are still in the infancy stage.

### Study Limitations

Despite the strengths of this trial, we anticipate some limitations. First, while the authors are ambitious to assume that the study centers are homogeneous, the heterogeneity among the 6 participating regional referral hospitals could cause bias and affect study estimates. Further, the relatively smaller number of clusters could affect the power of the study. However, randomization of 6 out of the 17 trauma centers is within the ethical constraints as this sample already represents 35.3% of Uganda’s regional referral hospitals. To overcome the statistical power bias limitation, the sample size is inflated for individual randomization to cater to the design effects in accordance with the report by Hooper et al [45]. Moreover, we plan to use mixed effects regression models, which permit adjustments for covariates at both the cluster and individual levels in a single-stage approach, and plan to use the *t* test, which is robust in terms of normality deviations. According to Borhan et al [52], mixed effects models are deemed suitable for smaller clusters in situations where the primary outcomes are continuous, which is the case in this trial. Further, we hope that the ancillary studies with mixed methods will add value to the overall interpretation of the trial results by providing data from individuals outside the health sector, such as traffic law enforcement professionals.

### Conclusion

Existing systematic reviews on trauma education programs in LMICs have identified a critical gap with regard to a lack of robust epidemiological studies that assess the effect of such interventions on patient outcomes within the low-resource context [16]. By relying on the strength of local needs contextualization to extend the training to nontrauma specialists, such as traffic law enforcement professionals, the results of this trial could inform the design, implementation, and scalability of rural trauma team development in similar low-resource settings. To the best of our knowledge, this is the first cluster randomized controlled trial using prospectively collected data to compare the effects of RTTDC training and standard care on patient outcomes and process measures in LMICs.

## Appendix

Multimedia Appendix 1 Informed consent form for rural trauma team development course participants.

Multimedia Appendix 2 Informed consent form for patient participants.

Multimedia Appendix 3 Outcome measurement variables and their analysis metrics.

Multimedia Appendix 4 SPIRIT (Standard Protocol Items: Recommendations for Interventional Trials) table showing the study timelines.

Multimedia Appendix 5 Patient participant data collection tool for the motorcycle trauma registry.

Multimedia Appendix 6 Trauma care frontline worker and public engagement trainee data collection tool.

Multimedia Appendix 7 Schematic representation of the study design.

## Abbreviations

CAC: cluster autocorrelation coefficient
CT: computed tomography
GCS: Glasgow Coma Scale
GOS: Glasgow Outcome Scale
ICC: intraclass correlation coefficient
ICU: intensive care unit
KTS: Kampala Trauma Score
LMIC: low- and middle-income country
MCQs: multiple-choice questions
RTTDC: Rural Trauma Team Development Course
TEFS: Trauma Expectation Factor Score
TOMS: Trauma Outcome Measure Score
WHO: World Health Organization

## Data Availability

The anonymized data sets arising from this study will be archived and made publicly available through a permanent weblink to a repository that will be provided by the publishing peer-reviewed journal.
