# Supplementary material for: Effect of Rural Trauma Team Development on the Outcomes of Motorcycle Accident–Related Injuries (Motor Registry Project): Protocol for a Multicenter Cluster Randomized Controlled Trial
Source: JMIR Res Protoc. 2024 May 7;13:e55297. doi: 10.2196/55297 (PMC11109866; doi:10.2196/55297)
Supplement: Multimedia Appendix 2 [file resprot_v13i1e55297_app2.docx]

**
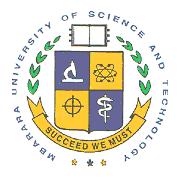
MBARARA UNIVERSITY OF SCIENCE AND TECHNOLOGY**

**RESEARCH ETHICS COMMITTEE**

P.O. Box 1410 Mbarara, Tel: +256-48-543-3795, Fax: +256-48-542-0782

E-mail: [irc@must.ac.ug](mailto:irc@must.ac.ug), mustirb@gmail.com

INFORMED CONSENT FORM (PATIENT PARTICIPANTS)

This document outlines the research study and expectations for potential participants. It should be written in layman terms and typed on MUST-REC letterhead.

**Instructions**

1. The wording of this document should be directed to the potential participant not MUST-REC.
2. If a technical term must be used, then define it the first time it is used, and any acronyms or abbreviations used should be spelled out the first time they are used.
3. All the sections of this document must be completed without any editing or deletions.
4. Please use a typing font that is easily distinguishable from the questions in this form. Preferably the font size should be 12.

**Study title** – This should be the same as on all other documents related to the study.

| **Lay title:** The use of allied health and law enforcement trauma team registries: Lessons from a novel motorcycle trauma registry and surveillance system in Uganda  **Scientific title:** An examination of the impact of rural trauma team development and training on the outcomes of motorcycle related orthopedic and neurological injuries: A protocol for a multi-center cluster randomized controlled clinical trial (The MOTOR trial) |
| --- |

**Principal Investigator(s)**

| Lule Herman |
| --- |

**Introduction**

What you should know about this study:

1. You are being asked to join a research study.
2. This consent form explains the research study and your part in the study.
3. Please read it carefully and take as much time as you need.
4. You are a volunteer. You can choose not to take part and if you join, you may quit at any time. There will be no penalty if you decide to quit the study.

**Brief background to the study**

| Limb and head injuries are a burden to passengers, motorcycle riders and people who walk on streets. The increasing number of motorcycle-related accidents could be a contributing factor. This study looks at the commonest injuries resulting from such accidents, the severity and outcome of such injuries and if forming teams of people who encounter such patients first especially in remote areas can help in improving the quality of information obtained, care for such injuries and their overall outcome. The study seeks to estimate the burden of motorcycle related accidents in our local context, contributing factors to poor outcome and contributing to management of such patients in this aspect. |
| --- |

**Purpose of the research project**

Include a statement that the study involves research, estimated number of participants, an explanation of the purpose(s) of the research procedure and the expected duration of the subject's participation.

This project is part of the requirements for an academic award and is expected to last for four years tentatively (2019 to 2023). We hope to recruit one to two patient participants per day in each of the participating hospitals. Each participant will be followed-up for a period of 3 months to assess how they are recovering and coping with their injuries. We shall also note any challenges your doctors might face in giving you treatment.

**Why you are being asked to participate?**

Explain why you have selected the individual to participate in the study.

You have been selected because this study is targeting patients involved in motorcycle-related accidents. If you have any injuries as result of a motor cyclist knocking another motorcycle, motor cyclist knocked by a car, you were walking on streets and knocked by a motorcycle or passenger on a motorcycle being involved in road traffic accident, you qualify for inclusion.

**Procedures**

Provide a description of the procedures to be followed and identification of any procedures that are experimental, clinical etc. If there is need for storage of biological (body) specimens, explain why, and include a statement requesting for consent to store the specimens and state the duration of storage.

If you are participating as a patient, your breathing, blood pressure, level of consciousness and serious injuries will be assessed.

You will be given emergency treatment like stopping the bleeding and pain killers based on your injuries until the doctor thinks you are stable before you can be enrolled into the study.

You will then be enrolled, asked a few questions regarding your age, profession, level of education, when and where the accident took place, how you arrived at the hospital and any care you received before hospital.

You will also be given a chance to tell us how you feel these injuries are going to affect your life and work today and 3 months from now. You will therefore be asked to provide a contact (for you and

Your attendant) for a curtsey phone call to find out how you are doing or remind you to come for your appointment and link or referral to any assistance you might need thereafter to recover from your injuries.

Once the doctor suspects you have a broken leg, or injured your head, you will be sent to “X-ray” or scan to confirm. This service is usually available freely in government facilities. The investigator will only meet this cost if the government free “X-ray” services are not available/functional. When you are ready to receive the results, your Drs will tell you the results and the next plan of management which the researcher will record on the questionnaire. You will however retain your “X-ray” film.

**Risks or discomforts**

Describe any reasonably foreseeable risks or discomforts-physical, psychological, social, legal, or other associated with the procedure, and include information about their likelihood and seriousness. Discuss the procedures for protecting against or minimizing any potential risks to the subject. Discuss the risks in relation to the anticipated benefits to the subjects and to society.

Apart from the discomfort that might arise from recalling the bad experience of events that occurred during the accident, there are no additional health risks posed by the study since it only requires you to respond to questions, undergo medical examination to detect any life threatening injuries that needs urgent attention and “X-ray” assessment (only if necessary) in accordance with trauma management guidelines to ascertain and confirm the nature of your broken leg or head damage.

**Benefits**

Describe any benefits to the subject or other benefits that may reasonably be expected from the research. If the subject is not likely to benefit personally from the experimental protocol note this in the statement of benefits.

As a participant, you will be linked to appropriate care of your injuries, access most recent information about the burden attributed to motorcycle related road traffic accidents and contribute to the existing body of knowledge in this field besides better understanding of the nature of your injuries. In the end, you will benefit when recommendations based on this study findings are implemented by policy makers, regarding prevention and improvement of case management of motorcycle injured patients

**Incentives or rewards for participating**

It is assumed that there are no costs to subjects enrolled in research protocols. Any payments to be made to the subject, e.g., travel expenses, token of appreciation for time spent, must also be stated, including when the payment will be made.

As a participant, you need to understand that your participation in this study is voluntary and that there is no economic gain from this study. You will get ten thousand shillings as compensation for your time and transport refund if your follow-up study visits are not related to routine patient care.

**Protecting data confidentiality**

Provide a statement describing the extent, if any, to which confidentiality or records identifying the subjects will be maintained. If data is in form of tape recordings, photographs, movies or videotapes, researcher should describe period of time they will be retained before destruction. Showing or playing of such data must be disclosed, including instructional purposes.

The investigator will protect all the information obtained with a password and lockable shelves with a key only known to him and will not reveal any of your personal information without your consent. Hard copies of information collected will be destroyed at the end of study period whereas anonymous soft copy will be archived.

**Protecting subject privacy during data collection**

Describe how the privacy of the participant will be ensured during the process of data collection.

Your name will never appear anywhere on any documents that will arise from this study, instead a unique number will be assigned to you. Only investigators will have access to this information

**Right to refuse or withdraw**

Include a statement that participation is voluntary and that refusal to participate will involve no penalty or loss of benefits to which the subject is otherwise entitled.

Since your participation is voluntary, nothing will happen if you refuse to participate in this study; or if you withdraw from the study.

**What happens if you leave the study?**

Include a statement that the subject may discontinue participation at any time without penalty or loss of benefits.

You will not incur any loss of benefits to which you would otherwise be entitled. You are free to withdraw from the study at any time without explaining your actions.

**Who do I ask/call if I have questions or a problem?**

Include contact for the researcher and Chairperson, MUST-REC.

If you have any queries/problem at any time about this study, contact Dr. Lule Herman C/o Turku Brain Injury Centre, Division of Clinical Neurosciences, Faculty of Medicine, University of Turku or reach him on Tel.0775656222/0758997877 or on E-mail address [lule.herman@gmail.com](mailto:lule.herman@gmail.com)

OR Contact Prof. Ssebuufu Robinson, Dean and Executive Director, Kampala International University-Western Campus P.o.Box 71-Ishaka on Tel: 0772507248 or E-mail [rssebuufu@gmail.com](mailto:rssebuufu@gmail.com)

OR Contact Dr. Francis Bajunirwe, Chairman Mbarara University of Science and Technology (MUST) IRC P.O.Box 1410 Mbarara Tel: 0485433795

**What does your signature or thumbprint on this consent form mean?**

Your signature on this form means

- You have been informed about this study’s purpose, procedures, possible benefits and risks
- You have been given the chance to ask questions before you sign
- You have voluntarily agreed to be in this study

_ _ _ _ _ _ _ _ _ _ _ _ _ _ _ _ _ _ _ _ _ _ _ _ _ _ _ _ _ _ _ _ _ _ _ _ _ _ _ _ _ _ _ _ _ _ _ _

Name of adult participant Signature of participant or Date

Legally authorized representative

_ _ _ _ _ _ _ _ _ _ _ _ _ _ _ _ _ _ _ _ _ _ _ _ _ _ _ _ _ _ _ _ _ _ _ _ _ _ _ _ _ _ _ _ _ _ _ _

Name of person obtaining consent Signature Date

_ _ _ _ _ _ _ _ _ _ _ _ _ _ _ _ _ _ _ _ _ _ _ _ _ _ _ _ _ _ _ _ _ _ _ _ _ _ _ _ _ _ _ _ _ _ _ _

Print Name of witness Signature or thumbprint or mark Date
