# Supplementary material for: Effect of Rural Trauma Team Development on the Outcomes of Motorcycle Accident–Related Injuries (Motor Registry Project): Protocol for a Multicenter Cluster Randomized Controlled Trial
Source: JMIR Res Protoc. 2024 May 7;13:e55297. doi: 10.2196/55297 (PMC11109866; doi:10.2196/55297)
Supplement: Multimedia Appendix 4 [file resprot_v13i1e55297_app4.docx]

**Multimedia Appendix 4.** SPIRIT (Standard Protocol Items: Recommendations for Interventional Trials) table showing the study timelines.

|  | **Law enforcement and medical trainee participant enrollment** | **Time of intervention** | **Patient participant**  **enrolment** | **Post-enrollment follow-up** | |
| --- | --- | --- | --- | --- | --- |
| **TIME POINTS** | 2 weeks prior each training session at each site | 2-days training every 3 months plus weekly audits | Day 1  (At Admission) | Day 2-7 until  Discharge/Referral | Day 90 |
| Eligibility screen | X |  | X |  |  |
| Informed consent |  | X (first day of the training) | X |  |  |
| Allocation | X (Cluster randomization of trauma centers at 3 weeks prior study commencement) | Team member allocation on first day of training | No individual patient allocation |  |  |
| **INTERVENTIONS:** | RTTDC Training Group | X |  |  |  |
|  | Control Group | No intervention |  |  |  |
| **ASSESSMENTS:** |  |  |  |  |  |
| a). Provider outcomes* |  |  |  |  |  |
| Pre-course MCQs score* |  | X (First day of the training) |  |  |  |
| Post-course MCQs score* |  |  |  |  | X |
| b). Patient outcomes |  |  |  |  |  |
| Baseline sociodemographic and clinical characteristics |  |  | X |  |  |
| Glasgow Coma Scale (GCS), |  |  | X |  |  |
| Trauma Expectation Factor Score (TEFS) |  |  |  | X |  |
| Glasgow Outcome Scale (GOS) |  |  |  |  | X |
| Trauma Outcome Measure Score (TOMS) |  |  |  |  | X |
| All-cause mortality |  |  |  |  | X |
| c). Process measure outcome |  |  |  |  |  |
| Pre-hospital interval (hours) |  |  | X |  |  |
| Referral-Exit Interval (hours) |  |  | X |  |  |
| Barriers to injury care* |  | X | X | X | X |
| *Additional measures for ancillary studies to the parent clinical trial | | | | | |
