# Supplementary material for: Effect of Rural Trauma Team Development on the Outcomes of Motorcycle Accident–Related Injuries (Motor Registry Project): Protocol for a Multicenter Cluster Randomized Controlled Trial
Source: JMIR Res Protoc. 2024 May 7;13:e55297. doi: 10.2196/55297 (PMC11109866; doi:10.2196/55297)
Supplement: Multimedia Appendix 6 [file resprot_v13i1e55297_app6.docx]

**PARTS 1 & 2 PRE-AND POST-RAINING QUESTIONNAIRE**

Reg. No…………………………………..Cadre/designation…………………………………….

Hospital where rotation/attachment is held……………………………………………………..

Instructions: (Circle the right answer for EACH of the MCQs 1-20)

1. When an injury occurs in a rural environment, there are many things that may cause a delay in the patient receiving definitive care. These delays result in an increase in preventable morbidity and mortality. Which of the following is NOT a potential delay that should be minimized in the rural environment?
2. Delay in the identification of immediately life-threatening injuries
3. Delay in the treatment prioritization of potentially life-threatening injuries
4. Delay in response time of the urban referral center
5. Delay in transport to higher level of care
6. Which of the following is INCORRECT?
7. The most important determinant of quality care is a timely, organized, rational response to the care of the trauma patient
8. Definitive care may not be possible or desirable in the rural environment
9. If definitive care is NOT possible, an attempt at diagnosing all injuries is not helpful if it results in a delay in transfer to an appropriate level of care
10. Pharmacy, blood bank, and radiology support services are always available in the rural environment
11. A 65-year-old woman is being brought to the emergency department (ED) of a rural facility by Emergency Medical Services (EMS) following a fall down a flight of stairs. Upon arrival, she has a seizure and then becomes unresponsive. To be most effective, the team leader in this situation must do all of the following EXCEPT:
12. Assign tasks/responsibilities
13. Articulate the plan
14. Personally execute all aspects of the plan
15. Adapt the plan on changes in the situation
16. A two-year-old (13 kg) girl is in the ED following a fall through a second-story screen window. She has normal vital signs (age appropriate) but has obvious deformities of both forearms. Prior to reducing and splinting these fractures, the team leader has asked that that patient be given 4 mg of morphine and 2 mg of midazolam intravenously. The nurse is concerned that these amounts may be too much medication. The nurse should:
17. Give the medication as ordered and carefully monitor the patient’s respiratory status
18. Clearly articulate the concern to the team leader in a respectful fashion
19. Report the medication error to the nursing supervisor
20. Secretly give half of the ordered amount and observe the patient’s reaction
21. Absolute indications for intubation include all the following EXCEPT:
22. T3 spinal cord injury
23. Glasgow Coma Score (GCS) of 8 or less
24. Severe maxillofacial injury
25. Neck injury with soft tissue swelling and airway compromise
26. Rapid sequence intubation:
27. Should be done only after the cervical spine is cleared of injury
28. Uses a long-acting paralytic such a pancuronium so that many attempts are possible
29. Is used to help assist with planned placement of a rescue airway device
30. Should be done following initial airway management with a bag-valve mask to allow preoxygenation
31. An 85-year-old female is transferred by ambulance to a 25-bed critical care access hospital with no surgical capability. The patient was found by a neighbor near a mailbox after she reportedly fell on a patch of ice. The exact time the patient was down on the ground is unknown. She is unresponsive to verbal and painful stimuli. Her heart rate is 50, blood pressure is 170/80, and she has respirations of 16. The nearest trauma center with neurosurgical capability is one hour away by fixed-wing aircraft. Prior to transfer, how should the airway be managed?
32. Does not need any airway intervention
33. Endotracheal intubation
34. Cricothyroidotomy
35. Supplemental oxygen using nasal cannula
36. All of the following injuries must be identified and immediately addressed during the primary survey EXCEPT:
37. Simple pneumothorax
38. Massive hemothorax
39. Tension pneumothorax
40. Open pneumothorax
41. A 25-year-old male was involved in a high speed, single vehicle crash. He is brought to a 25-bed, critical care access hospital with no surgical capability. His blood pressure is 70/30, heart rate is 135, respiratory rate is 45, and he is unresponsive to verbal stimuli. An emergency medical technician (EMT) is manually ventilating the patient with a bag valve mask. The patient’s neck veins are flat with no associated tracheal deviation. Initial auscultation of the chest reveals absent breath sounds on the right along with dullness to percussion. There is no obvious penetrating trauma. The diagnosis related to the thorax is:
42. Open pneumothorax
43. Flail chest
44. Tension pneumothorax
45. Massive hemothorax
46. A tension pneumothorax may be rapidly fatal. Clinical signs and symptoms of this include all of the following EXCEPT:
47. Muffled heart tones
48. Respiratory distress
49. Distended neck veins
50. Unilateral absence of breath sounds
51. The most common cause of shock in the injured patient is:
52. Cardiogenic
53. Hemorrhagic
54. Neurogenic
55. Septic
56. A 40-year-old man was involved in a motorcycle crash in which he was not wearing a helmet. His GCS is 3, heart rate is 120, and blood pressure is 90/60. There is external blood loss from his scalp. The volume of blood loss to cause shock in this adult patient can be found in all of the following locations EXCEPT:
57. External hemorrhage
58. Intrathoracic hemorrhage
59. Pelvic hemorrhage
60. Intracranial hemorrhage
61. Which of the following is TRUE regarding compensated shock?
62. Cardiac output is normal
63. Organ perfusion and tissue oxygenation is decreased
64. Hypotension is always present
65. Tachycardia is a sensitive sign in all elderly patients
66. Providers at a rural hospital without surgical resources must:
67. Determine the cause of shock before initiating the transfer process
68. Repair all lacerations before transfer
69. Obtain radiographs and treat all fractures before transfer
70. Initiate the transfer process when you suspect the patient has injuries that exceed your local resources
71. Secondary brain injury:
72. Refers to damage that occur shortly after injury
73. Does not influence overall outcome
74. Can be minimized by maintaining oxygenation and blood pressure
75. Is the same as spinal shock
76. A 32-year-old woman has a GCS of 8 after a fall down a flight of stairs. Her blood pressure is 85/60 and heart rate is 120. Her treatment must include:
77. Hypotonic fluid resuscitation
78. Mannitol
79. Hyperventilation
80. Endotracheal intubation
81. It is a warm summer afternoon. A 58-year-old male pedestrian was struck by a motor vehicle an hour ago and is now being assessed in your rural hospital emergency department. He is tachycardic with a blood pressure of 90/60, is restless and somewhat uncooperative, and is being resuscitated with crystalloids. Which of the following statements is correct?
82. Hypothermia is not a significant concern in this patient
83. Oxygen saturation measurements will be highly reliable in this individual
84. Room temperature should be optimized for trauma team members’ efficiency and comfort
85. Oral temperature measurements in this patient may not be reliable
86. You are assessing a 24-year-old woman in your community hospital emergency department. She was thrown from her snowmobile. Your team suspects a closed head injury, pelvic trauma, and a fractured femur. All of the following statements regarding her resuscitation are true EXCEPT:
87. The patient’s wet clothing should be removed
88. The receiving area should be warmed to 80 degrees Fahrenheit (27 degrees Celsius) prior to her arrival
89. Packed red cells should be placed in a microwave oven briefly before administration
90. Passive rewarming (dry blankets, Mylar sheets, plastic wrap) should be used
91. A Broselow Tape is:
92. Used to secure an endotracheal tube
93. A special adhesive used to secure cricothyroidotomy tube
94. Used to determine pediatric drug dosing
95. Used to assure inline stabilization of the neck for intubation
96. Children are less prone to rib fractures and mediastinal trauma and more prone to pulmonary parenchymal injuries because:
97. Children are rarely involved in traumatic incidents
98. Their lungs are less compliant and do not resist injury as well
99. Children have a compliant chest wall and mobile mediastinum
100. Children’s ribs are stronger and therefore resist fracture

The pre and post course questionnaire is with permission, based on educational materials of rural trauma team development course 4^th^ edition of the American College of Surgeons

**PART 3: BARRIERS TO INJURY CARE**

**Please rank the following delays as barriers to timely provision of definitive injury care for trauma patients in general. For each question, please use each numeric number once.**

1. In your current hospital of attachment, please rank the potential significant **system delays** to providing care to the injured (trauma) patients, in order of importance from the most important (score of 6) to the least important (score of 1) by assigning numbers 1, 2, 3, 4, 5, 6
2. Delays due to Discovery of the injured in the field
3. Delays in summoning/calling for help
4. Delays in mobilizing emergency medical service (EMS)/ambulance providers
5. Delays due to pre-hospital response time (ambulance arrival to scene)
6. Prolonged scene time (Delay at the scene)
7. Long transportation time to the hospital
8. Other (specify)……………………………………………………………………………
9. After the trauma patient has arrived to your current hospital of attachment, please rank the potential significant **team delays** to providing care to such patient in order of importance (by assigning a score of 4 to the most significant and 1 to the least significant (use 1,2,3,4)
10. Delays in identification of immediately life-threatening injuries
11. Delays in prioritization of life-threatening injuries
12. Delays in recognition of injury severity
13. Delays in transportation to a higher level of care
14. Other (specify)…………………………………………………………………………
15. In your current hospital, after the decision has been made to carry out emergency surgery for a trauma patient (for example amputation for a crushed limb), please rank the potential significant **barriers** to providing surgery to such patient in order of importance (by assigning a score of 4 to the most significant and 1 to the least significant. (use numbers 1,2,3,4)
16. Lack of skilled staff (surgical or anesthetic) to carry out the surgery
17. Lack of theatre space/functional theatre to carry out the surgery
18. Lack of supplies such as oxygen, emergency drugs, blood products, sutures
19. Lack of intensive and critical care (ICU) services for post-operative management
20. Other (specify)……………………………………………………………… …

**PART 4: PARTICIPANT COURSE EVALUATION FORM**

Instructions: This form provides an opportunity for you to rank each of the individual topics by ticking the appropriate box based on your overall individual experience and what you think of this kind of training.

| **S/N** | **Topics** | **Strongly Agree(5)** | **Agree (4)** | **Neutral(3)** | **Disagree (2)** | **Strongly Disagree (1)** |
| --- | --- | --- | --- | --- | --- | --- |
| 1 | Overall, this educational activity was excellent |  |  |  |  |  |
| 2 | Program topics and content met the stated objectives |  |  |  |  |  |
| 3 | Content was relevant to my educational needs |  |  |  |  |  |
| 4 | Educational format was conducive to learning |  |  |  |  |  |
| 5 | Acquired knowledge will be applied in my practice environment |  |  |  |  |  |
| 6 | I will seek additional information on this subject |  |  |  |  |  |
| 7 | Program was fair, objective, and unbiased towards any product or program |  |  |  |  |  |
| 8 | Power point slides are well written, visually appealing with good references |  |  |  |  |  |
| 9 | The audiovisuals enhance the presentation |  |  |  |  |  |
| 10 | Course format (lecture/ skill station scenarios) stimulates critical thinking |  |  |  |  |  |
| 11 | Content is organized in a concise and logical sequence |  |  |  |  |  |
| 12 | Instructor has knowledge about content |  |  |  |  |  |
| 13 | Instructor presentation style keeps learner’s attention |  |  |  |  |  |
| 14 | Instructor uses examples to illustrate major points |  |  |  |  |  |
| 15 | Instructor presents content accurately and confidently |  |  |  |  |  |
| 16 | Instructor answers questions in a supportive manner |  |  |  |  |  |
| **For each of the performance improvement/patient safety, communication & scenarios, rank the relevance of each module by ticking the corresponding box in the shaded area** | | | | | | |
| Rank (score) | | Performance improvement & patient safety | | Communication module | | Case scenarios |
| 1 | Not relevant |  | |  | |  |
| 2 | Relevant |  | |  | |  |
| 3 | Very relevant |  | |  | |  |

Adapted with permission from: Ali J, Kumar S, Gautam S, Sorvari A, Misra MC. Improving Trauma Care in India: the Potential Role of the Rural Trauma Team Development Course (RTTDC). Indian J Surg. 2015;77:227–31.
