# Supplementary material for: Effect of Rural Trauma Team Development on the Outcomes of Motorcycle Accident–Related Injuries (Motor Registry Project): Protocol for a Multicenter Cluster Randomized Controlled Trial
Source: JMIR Res Protoc. 2024 May 7;13:e55297. doi: 10.2196/55297 (PMC11109866; doi:10.2196/55297)
Supplement: Multimedia Appendix 7 [file resprot_v13i1e55297_app7.docx]

**Multimedia Appendix 7.** Schematic representation of the study design.

| Study duration: Three months for each period | | | | | | | | | | | | | | |
| --- | --- | --- | --- | --- | --- | --- | --- | --- | --- | --- | --- | --- | --- | --- |
| Study periods | | | 1 | 2 | 3 | 4 | 5 | 6 | 7 | 8 | 9 | 10 | 11 | 12 |
| Cluster | Control Group | A |  |  |  |  |  |  |  |  |  |  |  |  |
|  |  | B |  |  |  |  |  |  |  |  |  |  |  |  |
|  |  | C |  |  |  |  |  |  |  |  |  |  |  |  |
|  | Intervention Group | D |  |  |  |  |  |  |  |  |  |  |  |  |
|  |  | E |  |  |  |  |  |  |  |  |  |  |  |  |
|  |  | F |  |  |  |  |  |  |  |  |  |  |  |  |
| Study periods | | | 1 | 2 | 3 | 4 | 5 | 6 | 7 | 8 | 9 | 10 | 11 | 12 |
